# Dental caries in children and adolescents with poorly-controlled diabetes: a case-control study

**DOI:** 10.3389/froh.2024.1401485

**Published:** 2024-07-05

**Authors:** Shahd ElBshari, Imrana Afrooz, Rasha Hassan Beck, Rama Watad, Nabras Al-Qahtani, Asma Deeb

**Affiliations:** ^1^ Dental School, Ajman University, Ajaman, United Arab Emirates; ^2^ Paediatric Endocrine Division, Sheikh Shakhbout Medical City, Abu Dhabi, United Arab Emirates; ^3^ Clinical Trial Unit, Sheikh Shakhbout Medical City, Abu Dhabi, United Arab Emirates; ^4^ Medical School, Gulf University, Ajaman, United Arab Emirates; ^5^ College of Medicine and Health Sciences, Khalifa University, Abu Dhabi, United Arab Emirates

**Keywords:** adolescent, children, dental caries, diabetes, DMFT, DMFS, filling

## Abstract

The relationship between diabetes and dental caries remains uncertain. The main objective of this study was to quantify dental caries in children and adolescents with and without poorly-controlled diabetes to examine whether poorly-controlled diabetes influences caries prevalence and severity. This was a case-control study of children and adolescents with poorly-controlled diabetes and age-matched controls attending paediatric clinics at Sheikh Shakhbout Medical City, Abu Dhabi, UAE in August 2022. Dental caries was diagnosed by visual examination and dental probing to derive total number of decayed, missing, and filled tooth or surface (DMFT/DMFS) indices. Differences in caries metrics between subjects with diabetes and controls were assessed using chi-squared or Mann Whitney *U*-tests. Fifty-seven children and adolescents without diabetes and 42 with poorly-controlled (HbA1c ≥ 7.5) diabetes were recruited. The median (interquartile range, IQR) DMFT index was 4 (5) and the DMFS index was 4 (11). There were no significant differences in DMFT % [14.0 (21.5) vs.13.0 (20.0); *p* = 0.602], DMFT index [4 (5) vs. 3 (6); *p* = 0.749], nor DMFS index [5 (12) vs. 4 (11); *p* = 0.484] between patients and controls. Diabetes either has no effect on caries risk or its effect is so small that it is masked by dominant risk factors such as diet and obesity that require addressing through robust public health measures. While poor glycaemic control does not appear to influence caries risk, diet and obesity remain serious and addressable risk factors affecting oral health.

## 1 Introduction

Dental caries remains the most common chronic disease of childhood (1). Increased awareness of the importance of oral hygiene and the introduction of fluoride-containing toothpaste (2) have decreased the prevalence of the disease globally in children aged 5–12 years over the last four decades (1). Nevertheless, caries remains a significant public and national health problem in every country, including the United Arab Emirates (UAE) (3), where its prevalence in children and adolescents remains persistently high [at least 22%, but usually much higher in assessed populations (3)], despite advances in dental health provision and preventative education in the country. Identifying populations at risk of caries remains an important goal of paediatric dentistry, medicine, and public health, since the disease not only impacts the physical, psychological, and social wellbeing of young people but also their future risk of caries in adulthood and consequent health and financial costs (3).

The exact relationship between diabetes and dental caries risk remains uncertain in both children and adults. In adults, alterations in salivary secretions, such as in flow and glucose content (4), may predispose to caries lesions, and a few studies in children have detected alterations in pH, salivary flow rate, glucose, urea, total protein content, and bacterial composition in diabetic patients compared with healthy controls that could be cariogenic (5–7). Nevertheless, the epidemiological association between diabetes and caries risk in children and adolescents remains controversial, with some studies showing significantly higher [e.g. (8–11)], the same [e.g. (12–16)], or even lower [e.g. (17–19)] caries prevalence in patients with type 1 diabetes than in non-diabetic controls. Despite a majority of papers reporting no significant difference in caries lesions between type 1 diabetics and controls (20), relatively recent meta-analyses have detected significantly higher caries prevalence in both paediatric patients with type 1 diabetes (20) and adults with both type 1 or type 2 diabetes (21) compared with healthy controls.

The relationship between diabetes and dental caries is therefore likely to be complex, and it is important to establish associated risk in specific populations where local factors may influence the disease trajectory. Given that both diabetes and dental caries are common in UAE, we compared the dental caries status of children and adolescents with and without diabetes in Abu Dhabi, UAE. Since poor glycaemic control may increase the risk of dental caries (22), we selected patients with poor glycaemic control [ISPAD Clinical Practice Consensus Guidelines 2022 recommend an HbA1c target of <7.5 (23)] to increase the chances of detecting a significant influence of diabetes on caries prevalence and severity.

## 2 Materials and methods

This study is reported according to the Strengthening the Reporting of Observational Studies in Epidemiology (STROBE) guidelines for observational studies (24). This was a prospective observational, case-control study of children and adolescents with diabetes and age-matched controls without any metabolic abnormalities consecutively attending paediatric clinics at Sheikh Shakhbout Medical City (SSMC), Abu Dhabi, UAE in August 2022. The Institutional Review Board of the SSMC approved the study protocol (protocol number SSMCREC340), and all children or their parents/guardians provided written informed consent or assent for study participation.

All participants were children and adolescents aged ≤18 years. Inclusion criteria for patients with no diabetes were never diagnosed with diabetes or a chronic systemic illness. Patients with diabetes were classified according to WHO criteria (25). Patients with diabetes were on insulin pump therapy or were taking multiple daily injections (MDI) of insulin in a basal bolus regimen of insulin analogues. Patients with type 2 diabetes were managed with lifestyle modifications and metformin at a dose of between 1 and 2 g daily. All patients on insulin pump therapy and majority on MDI used continuous glucose monitoring; only a small number of patients use glucometers for self-monitoring of blood glucose. Exclusion criteria for all participants were if they were unable to provide informed consent or assent, cooperate with the study protocol, if data were missing or incomplete, or if they were at medical risk due to participation.

All children were examined by a dentist (IA) and a dental student (SE) in a dental chair under good illumination. Teeth were counted and classified into primary, mixed, or permanent dentition. Dental caries was diagnosed on tooth surfaces by visual examination and dental probing following WHO guidelines (26). No x-rays were taken. The WHO caries detection system has been widely used in epidemiological studies to assess caries prevalence in different populations and ages, and the DMFT/DMFS indices are defined as the total number of permanent teeth or surfaces that are decayed (D), missing (M), or filled (F) due to caries. The index can also be applied to the primary dentition (dmft/dmfs, in lower case), but here we denote the indices in capitals for convenience regardless of dentition status. The DMFT/DFMS scores and DMFT percentage (as a proportion of the number of teeth) were calculated. DMFT was graded as 0–1.1 (very low), 1.2–2.6 (low), 2.7–4.4 (moderate), 4.5–6.6 (high), and >6.6 very high, as previously (26).

Data are presented a medians (interquartile range; IQR) or counts (%) for continuous or categorical data, respectively. Differences in caries metrics between non-diabetic controls and diabetic patients were assessed using chi-squared tests or the Mann Whitney *U*-test for categorical and continuous data, respectively. Correlations between duration of diabetes and DMFT/DMFS were calculated using Spearman's rank correlations. All data were analysed in SPSS v.28 (IBM Statistics, Armonk, NY), and a *p*-value of <0.05 was considered statistically significant.

## 3 Results

### 3.1 Participant demographics and caries severity

The demographics of the study participants are shown in **Table 1**. Overall, 99 children and adolescents were recruited to the study, 56 (56.6%) of whom were female, with a median (IQR) age of 11.9 (5.5) years. The majority 57.6% of participants had their permanent dentition, while only 8.1% had their primary dentition alone. In the entire study population, the median DMFT index was 4 (5) (moderate), equating to a DMFT % of 14% (19.5%). Similarly, the DMFS index was 4 (11).

**Table 1:** Overall characteristics of the study participants.

Variable		No. (%)/median (IQR)
Sex	Male	43 (43.4)
	Female	56 (56.6)
Age		11.9 (5.5)
BMI standard deviation score		0.78 (0.64)
Type of diabetes (*n* = 39)	Type 1	36 (92.3)
	Type 2	3 (7.7)
Duration of diabetes (*n* = 39)		2.1 (6.1)
HbA1c (*n* = 39)		10.2 (3.9)
Total number of teeth		28 (4)
Dentition	Primary	8 (8.1)
	Mixed	34 (34.3)
	Permanent	57 (57.6)
DMFT %		14 (19.5)
DMFT index	Overall	4 (5)
	Primary	3.5 (7)
	Mixed	6 (5)
	Permanent	3 (4)
DMFS	Overall	4 (11)
	Primary	8 (16)
	Mixed	11 (13)
	Permanent	4 (6)

### 3.2 Caries severity in controls and patients with diabetes

Fifty-seven children and adolescents without diabetes and 42 children and adolescents with diabetes were recruited to the study, of whom 39 had complete data available. Of the young people with diabetes, 36 (92.3%) had type 1 diabetes and 3 (7.7%) had type 2 diabetes (Table 1), and the median time since diagnosis was 2.1 (6.1) years. Their median (IQR) HbA1c was 10.2 (3.9) and all HbA1c values were ≥7.5% (range 7.5–15.3%), indicating that this population had poorly-controlled disease.

The case and control groups were well matched in terms of sex, age, number of teeth, and dentition (all *p* > 0.05) (Table 2). There were no significant differences in DMFT % [14.0 (21.5) vs.13.0 (20.0); *p* = 0.602], DMFT index [4 (5) vs. 3 (6); *p* = 0.749], nor DMFS index [5 (12) vs. 4 (11); *p* = 0.484] between controls without diabetes and young people with diabetes, respectively. Similarly, there were no significant differences in DMFT and DMFS indices between groups for primary, mixed, and permanent dentition, with patients with mixed dentition showing the most severe (high and very high) median caries severity. There were also no differences in the median number of class 1 (occlusal surface) caries lesions between cases and controls (Mann Whitney *p* = 0.556). Finally, there were no correlations between duration of diabetes and caries severity (Spearman's rho −0.04 and 0.006 for DMFT and DMFS, respectively).

**Table 2:** Differences in dental health metrics between cases and diabetic patients.

Variable		Controls No. (%)/ median (IQR)	Diabetes No. (%)/ median (IQR)	*p*-value
Sex	Male	25 (43.9)	18 (42.9)	1.0
	Female	32 (56.1)	24 (57.1)	
Age		11.8 (6.3)	12.1 (4.9)	0.351
Total number of teeth		28 (4)	28 (4)	1.0
Dentition	Primary	5 (8.8)	3 (7.1)	0.941
	Mixed	19 (33.3)	15 (35.7)	
	Permanent	33 (57.9)	33 (57.1)	
DMFT index	Overall	4 (5)	3 (6)	0.749
	Primary	5 (7)	2 (-)	1.000
	Mixed	7 (4)	5 (6)	0.699
	Permanent	3 (3)	3 (5)	0.830
DMFT %		14.0 (21.5)	13.0 (20.0)	0.602
DMFS	Overall	5 (12)	4 (11)	0.484
	Primary	14 (15)	2 (-)	1.000
	Mixed	14 (15)	8 (13)	0.699
	Permanent	4 (5)	3.5 (7)	0.849
Class 1 lesions		2 (3)	3 (3)	0.556

## 4 Discussion

Using gold standard dental caries assessment according to DMFT/DFMS indices, we provide further evidence that diabetes does not adversely influence caries risk in young people. Given that previous evidence on the association between diabetes and caries risk and severity in children and adults has been contradictory, we only selected young people with poorly-controlled diabetes (HbA1c ≥ 7.5) to maximize the potential phenotypic effects of the disease on oral health. Despite this strategy, we did not detect any differences in caries metrics between patients with diabetes and metabolically normal controls, suggesting that diabetes either has no overall effect on developing dental caries or its effects are so small as to be masked by other dominant risk factors. Even though not formally included in ISPAD Consensus Guidelines (27), children with diabetes may also visit the dentist more frequently under instruction of their managing physician, thereby mitigating any adverse effects of the disease through improved oral healthcare.

Consistent with previous studies from the UAE and highlighting the national public health burden of dental caries (3), our population had an overall moderate severity of disease (DMFT scores between 2.7 and 4.4). Although difficult to directly compare our population with those sampled in other countries due to the age range of the study population (up to 18 years), our data suggest that the UAE has a major and persistent paediatric public health problem with dental caries, given that the median DMFT of 12-year-olds in other high-income countries is 1.3 (range 0.4–4.8) (1). While risk factors such as frequent snack consumption (28, 29), public school attendance (30), poor maternal education (31), and low socioeconomic status (32) may have an impact on caries risk and severity in children in the UAE, having a diagnosis of diabetes does not appear to affect the development or severity of caries in this age group. There is also poor dentist attendance (28, 33) and high levels of infrequent toothbrushing in the UAE: ∼30% of 5–6-year-olds reported skipping brushing at least once a month in one urban UAE population (Ajman) (29) and ∼15% of 4–6-year-olds did not brush at all in another urban UAE population (Ras Al-Khaimah) (33), consistent with Global School-based Student Health Survey (GSHS) data for the UAE, which showed that ∼20% of middle school students never or rarely brushed their teeth (34); again, diabetes does not appear to affect the development or severity of caries in this age group despite this risk. This is in contrast to studies of children in the UAE with some other systemic or chronic diseases in childhood including leukaemia (35), *β*-thalassemia (36), Down syndrome (37), or special healthcare needs (38), where DMFT values are significantly higher in affected populations than controls.

There is good evidence that diabetes has measurable effects on salivary flow and composition that may exert a pro-cariogenic effect (4–7), so the hypothesis that diabetes is a risk factor for dental caries is valid. This may be especially true for surfaces exposed to stagnant saliva or microorganisms, such as the occlusal surfaces, but we did not detect any differences in caries indices between cases and controls. Furthermore, the majority of insulin-treated patients with type 1 and type 2 diabetes experience hypoglycaemic episodes [up to 95% in a UAE population when measured prospectively (39)], which are often managed by eating simple carbohydrates (e.g., sweets) that might be a risk factor for dental caries, especially if the hypoglycaemia is at night. The absence of a detectable difference in caries between diabetic patients and controls could be for several reasons. First, the biological effects of diabetes on the oral microenvironment may be masked by the more dominant effects of other risk factors, especially well established risk factors such as diet (40). Indeed, the prevalence of obesity in the UAE is extremely high, with the Global School Health Survey 2010 reporting that approximately 40% of school children in the UAE are overweight or obese and cross-sectional data showing that nearly a third of adolescents drink sugar-sweetened beverages every day (41). Furthermore, in an urban population of 5–6-year-olds in the UAE, about 25% were classified as having a high level of sugary snack consumption (29). Despite the dietary demands of diabetes, the poor glycaemic control in our population would suggest that diabetic young people in the UAE similarly struggle with potentially harmful societal trends and behaviours, although this population was not overweight overall. Second, our entire population had a relatively high caries burden, reflecting an overall poor level of oral health in the population that again may mask the effects of diabetes, regardless of metabolic control. Third, young people with diabetes are embedded in the healthcare system and receive regular follow-up, including dental checks. Given that the frequency of dental visits is negatively associated with the development of caries (28, 33), this may have mitigated any increased risk from the disease. Finally, although the overall frequency of hypoglycaemia was within the usual frequency for children with diabetes on insulin, our education team counsels patients on how to treat hypoglycaemia, and all patients are provided with glucose tablets and gluco-gel to treat hypoglycaemia. This reduces the need for reverting to the traditional sugary drinks and sweets that remain in the oral cavity for a long time to treat the hypoglycaemia episodes, mitigating the cariogenic risk of hypoglycaemia.

Overall, our data show that the UAE must persist in its public health efforts to combat the high prevalence of dental caries in the country. The Community Dental Service's national oral diseases preventive program “Dubai Smiles Healthy” and “Abu Dhabi Smiles” were two programs launched in schools and health centres to improve paediatric oral health in the UAE (3). Given that dental caries has a multifaceted aetiology related to other socioeconomic factors, these efforts require joined-up thinking with other efforts to combat the increasing prevalence of obesity in the country (42) and equitable access to dental services.

Our study has some limitations. This was a relatively small single-centre study conducted in one geographic location in the UAE, mainly serving urban patients, so the results may not be generalizable to other populations. Nevertheless, our study provides important insights into the current state of oral health in the paediatric population in the UAE and potential causative factors. The study was an observational case-control design that may not have controlled for every relevant variable, so there may have been unknown differences between the groups to account for the observed results. Furthermore, we did not record data on other variable that may have been of interest and that might have influenced caries outcomes, such as frequency of dentist visits, toothbrushing, and insulin requirements, nor did we assess other oral health parameters such as plaque, gingivitis, gingival bleeding, and calculus formation, which may have established other links between diabetes and paediatric oral health. If clinical data were missing or incomplete, we excluded patients from the analysis, and this may have introduced bias. Unfortunately, although our multidisciplinary team follow the ISPAD guidelines on diabetes management including the area related to nutrition, it is difficult to confirm that all patients were strictly adherent to the given advice, and we did not include dietary questionnaires or food diaries in the study.

## 5 Conclusions

Nevertheless, this is in the first study of the relationship between diabetes and dental caries conducted in the UAE. Our data suggest that diabetes either has no effect on caries risk or that its effect is small enough to be masked by other dominant risk factors that still require addressing at the population level to improve the overall oral health of the nation. Despite these results, children and adolescents with diabetes must still be managed as high-risk patients and should be given consistent preventive messages to ensure a balance in struck between optimum oral health and diabetes control, stressing the importance of preventative dental care.

## Data Availability

The original contributions presented in the study are included in the article/Supplementary Material, further inquiries can be directed to the corresponding author.
